# YqiC of *Salmonella enterica *serovar Typhimurium is a membrane fusogenic protein required for mice colonization

**DOI:** 10.1186/1471-2180-11-95

**Published:** 2011-05-09

**Authors:** Mariela C Carrica, Patricio O Craig, Víctor A García-Angulo, Andes Aguirre, Eleonora García-Véscovi, Fernando A Goldbaum, Silvio L Cravero

**Affiliations:** 1 Instituto de Biotecnología, CICVyA-INTA Castelar, Los Reseros y Las Cabañas s/n, Buenos Aires, Argentina; 2 Fundación Instituto Leloir (IIBBA-CONICET), Av. Patricias Argentinas 435, Buenos Aires, Argentina; 3 Instituto de Biología Molecular y Celular de Rosario, Consejo Nacional de Investigaciones Científicas y Técnicas, Departamento de Microbiología, Facultad de Ciencias Bioquímicas y Farmacéuticas, Universidad Nacional de Rosario, Suipacha 531, S2002LRK Rosario, Argentina

## Abstract

**Background:**

*Salmonella enterica *serovar Typhimurium is an intracellular bacterial pathogen which can colonize a variety of hosts, including human, causing syndromes that vary from gastroenteritis and diarrhea to systemic disease.

**Results:**

In this work we present structural information as well as insights into the *in vivo *function of YqiC, a 99-residue protein of *S*. Typhimurium, which belongs to the cluster of the orthologous group 2960 (COG2960). We found that YqiC shares biophysical and biochemical properties with *Brucella abortus *BMFP, the only previously characterized member of this group, such as a high alpha helix content, a coiled-coil domain involved in trimerization and a membrane fusogenic activity *in vitro*. In addition, we demonstrated that YqiC localizes at cytoplasmic and membrane subcellular fractions, that a *S*. Typhimurium *yqiC *deficient strain had a severe attenuation in virulence in the murine model when inoculated both orally and intraperitoneally, and was impaired to replicate at physiological and high temperatures *in vitro*, although it was still able to invade and replicate inside epithelial and macrophages cell lines.

**Conclusion:**

This work firstly demonstrates the importance of a COG2960 member for pathogen-host interaction, and suggests a common function conserved among members of this group.

## Background

*Salmonella enterica *is an intracellular facultative anaerobe Gram-negative that infects a variety of hosts, which include mammals, avians and reptiles. In human beings, *S. enterica *causes over 33 million cases of disease worldwide annually, which may vary from gastroenteritis and diarrhea to severe life-threatening systemic disease (typhoid fever) [1]. The outcome of the disease depends on both the serovar of *Samonella *and the host susceptibility. *Salmonella enterica *serovar Typhimurium (*S*. Typhimurium), can infect humans and animals, but causes different syndromes in each host. In humans, *Salmonella *produces enterocolitis, but in mice it causes a systemic illness that resembles human typhoid fever. Because of this, *S*. Typhimurium is widely used as a model organism to study the host-pathogen interactions that contribute to the onset of the systemic disease [2,3].

The pathogenic strategy of *S*. Typhimurium includes penetration of the mucosal barrier, invasion of non-phagocytic cells of the intestinal mucosa and survival and replication inside macrophages of the spleen and liver during the systemic phase. The ability of *S*. Typhimurium to survive to host defense mechanisms and to cause disease has been directly linked to genes encoded in pathogenicity islands, which are large horizontally acquired regions of the chromosome. Of outstanding importance are two type three secretion systems (T3SS), which together with cognate effector proteins are essential for cell invasion, intracellular survival and, therefore, for mice colonization [4]. Besides, factors encoded in the genomic backbone of *Salmonella *are also important for virulence in the murine model [5-8].

YqiC is a 99-residue protein of *S*. Typhimurium (UniProtKB entry K09806, gene STM 3196) which belongs to the cluster of orthologous groups 2960 (COG 2960). This COG includes 322 members (Pfam June 2010), encoded in genomes of pathogenic, non-pathogenic and symbiotic bacteria. In spite of the high conservation of this COG across bacterial species, no description of the in vivo function of any member has been reported. In this work, we carried out microbiological studies which demonstrate that YqiC is required for the pathogenesis of S. Typhimurium in the murine model, since a null mutant is highly attenuated when inoculated both orally and intraperitoneally. We also show that this protein is dispensable for cell invasion and intracellular replication in murine macrophages and human epithelial cell lines, but it is necessary for efficient growth at the mammalian host physiological temperature outside the cells. The microbiological results are complemented by biophysical and biochemical studies. These analyses demonstrate that YqiC shares properties with the recently reported BMFP from *Brucella abortus *(another member of the COG 2960) which include a trimeric coiled-coil structure and the ability to induce membrane fusion *in vitro *[9]. The results presented here contribute to elucidate the function of members of the COG 2960 and their biological role.

## Results

### *S*. Typhimurium YqiC is a trimeric protein with a high helical content

**Figure 1:**
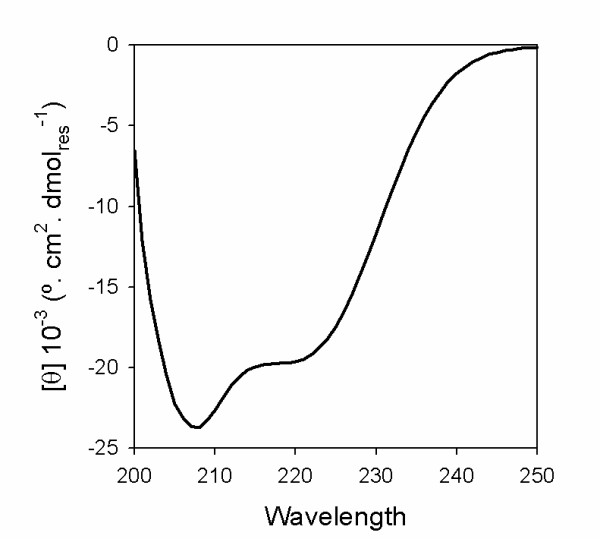
**Far UV-CD spectrum of YqiC measured in 50 mM Tris-HCl, 150 mM NaCl buffer (pH 8.0)**.

YqiC is a 99-residue protein of S. Typhimurium (UniProtKB entry K09806) which belongs to the cluster of orthologous groups 2960 (COG 2960). The bioinformatic analysis of the primary sequence of YqiC predicts a high helical content (66-77%) http://www.predictprotein.org, including two helical segments that span the N- and C-terminal halves of the protein (encompassing residues 4-43 and 49-79, respectively). Both helical segments are amphipathic but only the C-terminal one is predicted to form a coiled-coil structure http://groups.csail.mit.edu/cb/paircoil/paircoil.html. YqiC secondary structure was experimentally determined by its far UV circular dichroism spectrum (Figure 1), which showed a typical signature of an alpha helical protein. The percentage of helical structure of YqiC, estimated through the analysis of its CD spectra using K2D program (63%), agrees with the percentage of amino acids involved in the predicted N- and C-terminal alpha helices.

**Figure 2:**
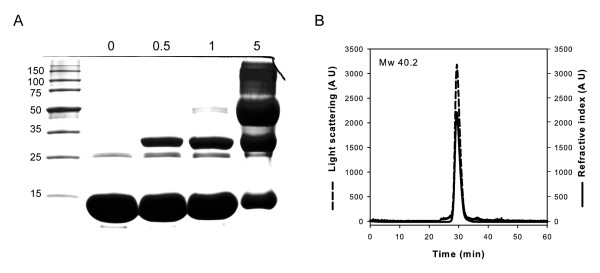
**Quaternary structure analysis of YqiC**. (A) Chemical cross-linking. Cross-linked products were separated via 15% SDS-PAGE followed by Coomassie brilliant blue staining. Protein markers are shown in kilodaltons. The numbers 0, 0.5, 1, and 5 indicate the millimolar concentrations of ethylene glycol bis (succinimidyl succinate) used. (B) Gel filtration coupled to SLS analysis. The protein was run on a Superdex-75 column and eluted with 50 mM Tris-HCl, 150 mM NaCl buffer (pH 8). The molecular mass of the protein was calculated relating its light scattering at 90° (dashed line) and refractive index (solid line) signals, and comparison of this value with that obtained for BSA as a standard.

On the other hand, we studied the oligomeric state of YqiC by chemical cross-linking and static light scattering. Chemical cross-linking of YqiC yielded trimers as the largest products when the amount of cross-linking reagent was increased (Figure 2A). Moreover, analysis of YqiC by static light scattering coupled to size exclusion chromatography showed a single homogeneous peak with a molecular mass of 40.2 kDa, in agreement with a trimeric structure (Figure 2B).

The characteristics described here are similar to the structural features that we have previously reported for *Brucella abortus *BMFP, which is a member of the COG 2960 that only conserves 22% sequence identity with YqiC [9].

### YiqC promotes membrane fusion in vitro

**Figure 3:**
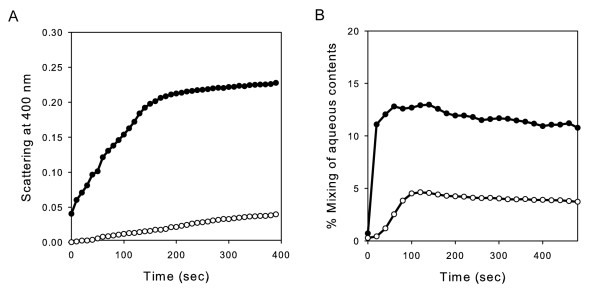
***In vitro *liposome aggregation and fusion induced by YqiC**. (A) Time course of DPPC/DPPA SUV aggregation monitored by light scattering and (B) time course of aqueous content mixing was measured after addition of YqiC protein. Equimolar amounts of terbium (Tb)- and dipicolinic acid (DPA)-loaded SUV were premixed in 10 mM Tris-HCl (pH 8.0), 50 mM NaCl, and 1 mM EDTA. The fluorescence of the Tb(DPA)3 complex formed after the mixing of aqueous contents by protein addition was measured at 545 nm over incubation time. The measurements were taken in 50 mM Tris-HCl buffer (pH 8.0) (open circles) or 50 mM sodium acetate buffer (pH 4.0) (close circles) at 25°C. The liposomes were composed of DPPC and DPPA in a molar ratio of 75:25. The lipid:protein molar ratio was 100: 1. The data presented are the results of a representative experiment of three independent repetitions.

As YqiC shares structural properties with BMFP, we investigated if this protein also conserves the membrane fusion activity reported for BMFP [9]. With this aim, we measured the increase in the size and aqueous content mixing of phospholipids vesicles produced after YqiC addition. Changes in the size and aggregation state of vesicles were evaluated by turbidity measurements at 400 nm whereas the aqueous content mixing was evaluated by measuring the fluorescence of the Tb-DPA complex produced upon fusion of vesicles containing TbCl_3 _or DPA encapsulated in their aqueous interior phase, and the percentage of mixing was calculated as described in materials and methods. Experiments were carried out on small unilamellar vesicles composed of a mixture of DPPC and DPPA in a 75:25 molar ratio, both at acid or neutral pH. YqiC produced both a significant increase in the turbidity (Figure 3A) and aqueous content mixing (Figure 3B) in the vesicle solutions, mainly at acid pH, after addition of YqiC. These results indicate that YqiC has a pH-dependent in vitro fusogenic activity.

### Subcellular localization of YqiC

**Figure 4:**
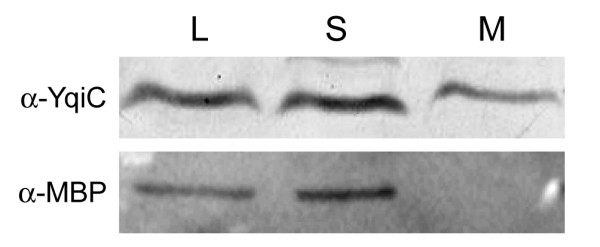
**Subcellular localization of YqiC**. Whole-cell lysate of S. Typhimurium was fractionated by ultracentrifugation. Samples of the cell lysate (L), the supernatant (S) and the sedimented membrane fraction (M) were analyzed by immunoblotting with anti-YqiC and anti-MBP antiserum. Antibodies against the soluble MBP protein [10] was used as a control for the membrane fraction contamination.

To determine the subcellular localization of YqiC, we performed a mechanical lysis fractionation procedure. A wild type *S*. Typhimurium culture grown to late log phase was harvested by centrifugation, mechanically disrupted and fractionated by ultracentrifugation. This procedure allows for the separation of bacterial proteins into two fractions: the supernatant, which contains cytoplasmic and periplasmic proteins, and the pellet fraction, which contains the inner and outer membrane proteins. Fractions were then analyzed by immunoblotting using an anti-YqiC polyclonal antibody. YqiC was localized in the two fractions, although lower levels of YqiC were found in the membrane fraction (Figure 4). This result indicated that YqiC is both soluble and membrane associated inside the cell. As a control, we used an antibody against the periplasmic protein MBP [10], which was only detected in the supernatant fraction.

### Evaluation of a *yqiC *defective strain phenotype in vitro

**Figure 5:**
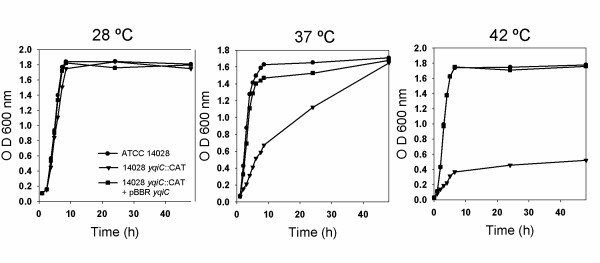
**Growth curve of S. Typhimurium ATCC 14028 (circles), 14028 Δ*yqiC*::CAT (triangles), and 14028 Δ*yqiC*::CAT + pBBR *yqiC *(squares) at different temperatures**. A 1:50 dilution of a saturated culture in LB was incubated at 200 rpm, at the indicated temperature. The OD_600 _was measured at different time points over 48 hours. The data presented are the results of a representative experiment of three independent repetitions.

The *in vivo *functions of the members of the COG 2960 are unknown. To investigate the role of YqiC protein in S. Typhimurium, we constructed an *S*. Typhimurium ATCC 14028 null mutant in *yqiC *through allelic exchange. The resulting strain was named 14028 Δ*yqiC*::CAT. The gene *yqiC *is encoded divergently to the *ribB *gene and convergent to the *glgS *gene in the *S*. Typhimurium chromosome. Thus, it appears that *yqiC *is transcribed as a monocistronic element, and polar effects upon allelic exchange are not expected. The successful elimination of the *yqiC *gene was corroborated by PCR analysis and a western blot assay of cell lysates of 14028 Δ*yqiC*::CAT and its complemented derivative (bearing plasmid pBBR-*yqiC*, which encodes intact *yqiC *gene), using a polyclonal antibody raised against YqiC (data not shown). As a first approach to assess the effect of the mutation in the physiology of *Salmonella*, we tested the effect of temperature in the replication of *yqiC *mutant strain in LB. No difference in the growth pattern of the *yqiC *mutant strain compared with the WT was detected at 28°C (average generation time 44.9 +/- 1.4). However, an increased generation time at 37°C was observed for 14028 Δ*yqiC*::CAT, where the average generation time was 22.5 +/- 0.7 minutes for S. Typhimurium 14028 and 48 minutes for 14028 Δ*yqiC*::CAT (Figure 5). This difference in growth was enhanced when the strains were incubated at 42°C, where the average generation time was 30.2 +/- 0.68 minutes for the WT strain and 78.9 +/- 0.7 minutes for the Δ*yqiC*::CAT mutant strain. At both temperatures, *trans *complementation with the plasmid encoding *yqiC *restored the wild-type growth curve pattern to 14028 Δ*yqiC*::CAT. These results indicate that the mutation of *yqiC *affects the ability of *S*. Typhimurium to replicate at physiological and high temperatures. No growth curve pattern alteration was observed for the 14028 Δ*yqiC*::CAT strain when incubated in M9 minimal media or acid LB (pH = 4.0) at 28°C (data not shown), which indicates that the *yqiC *mutant is neither auxotrophic nor acid sensitive.

### Survival of the STM-*yqiC*mutant in cultured cells

**Figure 6:**
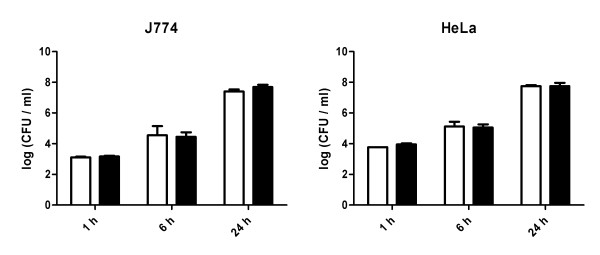
**Invasion and intracellular survival of *S*. Typhimurium strains in cultured cells**. *S*. Typhimurium ATCC 14028 (open bars) and 14028 Δ*yqiC*::CAT mutant (filled bars) recovered from lysates of J774 murine macrophages (A) or human epithelial HeLa cells (B). The number of viable bacteria from cell lysates was determined 1, 6 and 24 hours post infection as described in *Materials and methods*. The reported value is the media of duplicates of a representative experiment +/- standard deviation.

The pathogenicity of *S*. Typhimurium is critically dependent on its ability to infect and multiply into eukaryotic cells. We investigated whether the 14028 Δ*yqiC*::CAT strain was affected in its ability to invade and survive within cultured eukaryotic cells. J774 murine macrophages and HeLa human epithelial cell lines were infected with WT *S*. Typhimurium and 14028 Δ*yqiC*::CAT strains. As the 14028 Δ*yqiC*::CAT strain grows defectively at physiological temperature, all strains were grown at 28°C prior to infection. Infected cells were kept at 37°C and viable intracellular bacteria was determined in cell lysates at 1, 6 and 24 hours after infection. In both cell types, no differences were detected at all time points examined in the CFU recovered from cell lysates infected with the WT or the *yqiC *mutant strains (Figure 6). This result indicates that the *yqiC *gene does not contribute to neither *Salmonella *entry nor intracellular survival in the cell types assayed.

### Role *of S*. Typhimurim YqiC in virulence

**Figure 7:**
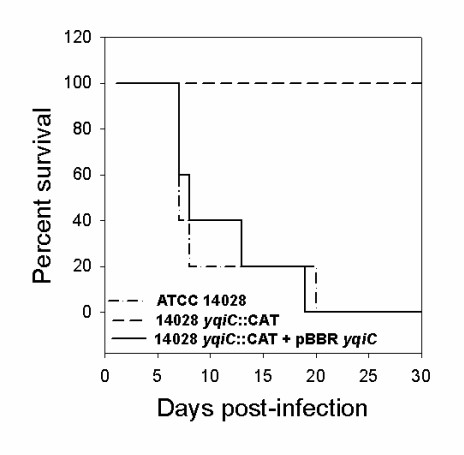
***yqiC *is essential for virulence in mice**. BALB/c mice were orally infected with 1 × 10^5 ^CFU of wild-type *S*. Typhimurium ATCC 14028, 14028 Δ*yqiC*::CAT or 14028 Δ*yqiC*::CAT + pBBR-*yqiC*. The survival of infected mice over time is shown.

In spite of the clear effect of the *yqiC *mutant strain on growth at 37°C, we did not observe any defect in colonizing and surviving inside *in vitro *cultured eukaryotic cells grown at 37°C. Thus, we evaluated the virulence of the *yqiC *mutant in the murine model. To this aim, we performed oral infections with *S*. Typhimurium ATCC 14028, 14028 Δ*yqiC*::CAT and 14028 Δ*yqiC*::CAT *trans*-complemented with *yqiC *in BALB/c mice. As illustrated in Figure 7, no survival was registered by day 20 in mice inoculated with WT strain. However, the *yqiC *mutant showed complete attenuation in virulence, as all mice infected with this strain survived along the 30-day period of the experiment. The *yqiC *gene provided in *trans *fully complemented the 14028 Δ*yqiC*::CAT phenotype, causing 100% mice death by day 19. In addition, we determined the LD50 of *S*. Typhimurium ATCC 14028 and 14028 Δ*yqiC*::CAT in mice inoculated intraperitoneally as described in *Materials and methods*. A dramatic increase in the LD50 was observed in the *yqiC *defective strain (>5 × 10^5 ^CFU), as compared with the WT (10-100 CFU) (Table 1). Together, these results clearly show that YqiC is required for *S*. Typhimurium virulence in the murine infection model.

**Table 1:** Determination of LD50 of *S*.Typhimurium strains in mice.

	Number of dead mice/Number of infected mice (Mean of days to death)	
Dose (CFU/mouse)	*S*. Typhimurium ATCC 14028	*S*. Typhimurium 14028 Δ*yqiC*::CAT
1 × 10^1^	3/7 (6)	0/7
1 × 10^2^	7/7 (6.7)	0/7
1 × 10^3^	6/6 (5.5)	0/6
1 × 10^4^	6/6 (4.5)	0/6
1 × 10^5^	6/6 (4)	0/6

Groups of the indicated number of mice were inoculated intraperitoneally with different doses of *S*. Typhimurium ATCC or S. Typhimurium strain and survival was recorded for up to 30 days.

## Discussion

In this work we have characterized the YqiC protein of S. Typhimurium. YqiC shares common structural and biochemical characteristics with its previously reported orthologous BMFP protein of *Brucella abortus *[9], although these proteins share only 22% of sequence identity and *Brucella spp *and *Salmonella *are phylogenetically distant bacteria. The common structural characteristics between YqiC and BMFP, namely high alpha helix content, coiled coil C-terminal and amphipathic alpha helix N-terminus, are also predicted by bioinformatics analysis for other proteins of the COG 2960 (such as those encoded by *Escherichia coli*, *Shewanella oneidensis*, *Legionella pneumophila*, *Xanthomonas campestris*, *Pseudomonas aeruginosa*, *Bordetella pertussis*, *Agrobacterium tumefaciens*, *Sinorhizobium meliloti *and *Rhodopseudomonas palustris*). This structural conservation strongly suggests a common function for the members of this COG.

In addition, we demonstrated that YqiC has membrane fusogenic activity, like BMFP and other trimeric coiled-coil and/or amphipathic proteins [11,12]. This activity is higher at acidic pH. A similar fusogenic activity at low pH was observed for *B. abortus *BMFP (unpublished data). The fusogenic activity could be relevant as many processes that involve bacterial or host cell membrane fusion events are important for pathogenic bacteria to successfully establish host infection. In this regard, both *S*. Typhimurium and *B. abortus *penetrate the cell by phagocytosis, and reside within a host vacuole that rapidly undergoes acidification. This drop in the pH serves as a signal for the expression of bacterial factors that alter intracellular membrane traffic in order to set their replicative niche [13-15]. The improved YqiC activity at low pH could indicate that this protein is active at the vacuolar stage of the bacterial infection. It is interesting to highlight that YqiC shares structural similarity with S. Typhimurium-SipB protein, as both are predominantly alpha helical in aqueous solution and have a coiled-coil domain involved in trimerization [16]. SipB is an effector protein essential for *Salmonella *invasion secreted through the SPI-1-encoded T3SS and was the first bacterial protein reported to display membrane fusogenic activity [16], however the function of this membrane fusogenic activity in the bacterial pathogenesis has not been clearly defined [17]. The activity of YqiC may be required during the interaction of *Salmonella *with the host cell to hijack membrane trafficking pathways. This would probably be accomplished by competitive inhibition, mimicking eukaryotic membrane fusogenic proteins, such as the SNAREs (given the structural similitude with these proteins) and inhibiting lysosomal fusion with the *Salmonella*-containing vacuoles. Current work is addressing whether YqiC is translocated to the host cell.

Alternatively, the YqiC-membrane fusogenic activity could be required during the biogenesis of bacterial outer membrane vesicles (OMV), which are spherical bilayered structures liberated from the outer membrane in Gram negative bacteria [18]. OMV act as delivery vesicles for bacterial toxins into host cells, promote quorum sensing, are involved in stress response, inhibit phagosome-lysosome fusion during bacterial growth within macrophages and are important constituents of the matrix of Gram-negative and mixed bacterial biofilm [19-23]. To date, the machinery that cause vesicle formation remains elusive but it may be expected that a protein with membrane fusion activity could be involved in this process [18,24]. In this regard, in spite of the lack of a signal peptide or transmembrane domains we demonstrated that YqiC can be localized both soluble and associated to membranes. This localization pattern was also observed for *B. abortus *BMFP (unpublished data). Subcellular localization pattern of YqiC may be in tune with its hypothetical function in biogenesis of OMV, as soluble and membrane-bound states of YqiC can be related to transient associations of this protein with the outer membrane. At this point, is interesting to note that OMV produced by *Shigella flexneri *contain IpaB, a SipB homologue which also displays membrane fusion activity [25,26]. Accordingly, many of the bacterial species conserving an YqiC homolog have been shown to generate OMV [18,27]. Further work is needed to investigate the possible role of YqiC in the biogenesis of OMV.

The in vivo importance of YqiC was demonstrated by the fact that this protein is necessary for *S*. Typhimurium virulence in the murine model, as an *yqiC *mutant strain was unable to kill mice within the period of time assayed and had a significantly higher LD50. The basis for this attenuation in virulence may be related to the observed defect to grow at physiological temperature *in vitro*. Temperature represents a common environmental challenge that microorganisms should be able to sense and respond to in order to survive [28]. Many other single gene mutations produce temperature-sensitive, virulence-attenuated *Salmonella *strains. Examples include *smpA*, which encodes for an outer membrane lipoprotein, *uspA*, which encodes for an universal stress response protein and the genes for DegP and DegQ proteases [29-31]. Interestingly, temperature sensitivity could not be the only factor responsible for the virulence attenuation observed for the *yqiC *mutant, as this strain was still able to invade and replicate inside macrophages and epithelial cell lines incubated at 37°C. These phenotypes may be due to differences in the metabolic status and environmental conditions affecting bacteria replication in rich media under laboratory conditions and inside the eukaryotic cell.

## Conclusion

We have demonstrated in this work that *S*. Typhimurium YqiC shares structural and biochemical characteristics with *B. abortus *BMFP, in spite of their relatively low sequence identity. Thus, members of the COG 2960 may accomplish a conserved function among phylogenetically distant bacteria. This function may be necessary to display full virulence. This seems to be the case, as in a parallel work we observed virulence attenuation when analyzing a *B. abortus *BMFP-defective strain (Cravero *et al*, unpublished work). This work is the first demonstration of the *in vivo *importance of a member of the COG 2960. However, future research is necessary to clarify the physiological processes in which the membrane fusogenic activity and possibly other unknown functions of YqiC are required.

## Methods

### Ethics Statement

All experiments involving animals have been approved by the ethics committee of the Instituto Nacional de Tecnologia Agropecuaria (INTA) where they were conducted. This ethics committee works according with the National Institutes of Health Guide for the Care and Use of Animals Laboratory [32].

### Bacterial Strains and Growth Conditions

For this study, we used the WT *Salmonella enterica *serovar Typhimurium strain ATCC 14028. Bacterial strains were grown in Luria-Bertani (LB) or M9 minimal medium containing casamino acids and glucose. Appropriate antibiotics were added to the following final concentrations: 100 *μ*g ml^-1 ^ampicillin, 25 *μ*g ml^-1 ^kanamycin, and 10 *μ*g ml^-1 ^chloramphenicol.

### DNA Manipulation and plasmid construction

Plasmid pET24D-YqiC (encoding full length YqiC protein) was generated by polymerase chain reaction (PCR) from *Salmonella enterica *serovar Typhimurium ATCC 14028 chromosome DNA using primers 5'-AACCATGGTTGACCCGAAAAAAATT-3' and 5'-TTCTCGAGCTCTTGTTGTGGATCGAC-3' and the product was cloned in *Nco*I and *Xho*I sites of pET24D vector (Novagen) in frame with the T7 promoter. The product included a six-histidine tag fused to the C-terminal end of the protein. To construct plasmid pBBR-*yqiC*, a 1210 bp fragment containing *yqiC *gene and flanking regions from *S*. Typhimurium was amplified by PCR using the primers 5'-GGCTTCAATGGTCACGGTAA-3' and 5'-GCAATATGGACGAGGAGCATC-3'. The resulting fragment was then cloned into the *EcoRI *site of the broad-host-range plasmid pBBR1MCS1 [33].

### Expression and Purification of Recombinant Protein

pET24D plasmid encoding the sequence of *yqiC *was transformed in *E. coli *BL21 (lambda DE3). The cells were grown in LB at 37°C to an OD 600 of 0.5 and induced with 1 mM isopropyl *β*-D thiogalactoside (IPTG) for 4 h. Cells were harvested by centrifugation at 3000 × *g *for 20 min, resuspended in binding buffer (Qiagen), and disrupted by sonication with a probe tip sonicator. Total cell lysate was centrifuged at 14000 × *g *for 30 min to remove non-soluble protein, cell debris, and unbroken cells. Binding and elution from nickel nitrilotriacetic acid-agarose resin were carried out under native conditions according to the manufacturer's instructions (Qiagen). Eluted proteins were dialyzed against phosphate-buffered saline (pH 7.4). Proteins were assayed with a Coomassie blue-based staining solution.

### Vesicle Preparation

Phospholipids were purchased from Avanti Polar Lipids (Birmingham, AL) and from Sigma. L-α-dipalmitoylphosphatidylcholine (DPPC) and L- α-dipalmitoylphosphatidic acid (DPPA) were cosolubilized in chloroform in different molar ratios, dried under N_2_, resuspended in buffer 50 mM Tris-HCl pH 8.0 or 50 mM sodium acetate pH 4.0 and sonicated to yield small unilamellar vesicles (SUV).

### Chemical Cross-Linking

Purified YqiC was cross-linked with ethylene glycol bis (succinimidylsuccinate) (EGS) (Sigma) used at concentrations of 0.5, 1.0, and 5.0 mM. The reactions were carried out for 30 min at room temperature in phosphate-buffered saline and stopped by addition of 50 mM Tris-HCl, pH 8.0. Cross-linked products were analyzed by SDS-PAGE.

### Determination of the Molecular Weight by Static Light Scattering

The average molecular weight (*M*w) of YqiC was determined on a Precision Detector PD2010 light scattering instrument tandemly connected to a high-performance liquid chromatography system and an LKB 2142 differential refractometer. The sample was loaded on a Superdex 75 column and eluted with PBS buffer. The 90° light scattering and refractive index signals of the eluting material were analyzed with Discovery32 software, supplied by Precision Detector. The 90° light scattering detector was calibrated using bovine serum albumin (66.5 kDa) as a standard.

### Circular Dichroism Spectroscopy

The circular dichroism (CD) spectra of YqiC in the far-UV region (250-200 nm) were measured on a Jasco J-810 spectrophotometer using quartz cuvettes with a path length of 0.1 cm. The CD spectra were analyzed with K2D software http://www.ogic.ca/projects/k2d2/[34] to evaluate the secondary structure content.

### Turbidity Assay

Turbidity measurements were taken on a Multiskan Spectrum double-beam spectrophotometer (Thermo Electro Corp.) by using 1 cm matched silica cuvettes at 400 nm. The SUV concentration was 250 *μ*M. The lipid:protein ratio for the turbidity assays was kept at 50:1.

### Vesicle Internal Content Mixing

*Small unilamellar *vesicles were prepared containing either 5 mM terbium chloride, 50 mM sodium citrate,10 mM Tris/HCl (pH 7.4), or 50 mM sodium dipicolinate (DPA) and 10 mM Tris-HCl (pH 7.4). The vesicles concentration was 100 μM. In both cases, no encapsulated material was removed by gel filtration of the vesicles using Sephadex G-25 (Pharmacia) equilibrated with iso-osmolar 50 mM NaCl, 1 mM EDTA, and 10 mM Tris-HCl (pH 7.4). Zero percent and 100% fluorescence (aqueous content mixing) were taken as the intrinsic fluorescence intensity of the Tb/DPA-labeled liposome mixture and the fluorescence obtained after vesicle lysis with 0.2% *n*-dodecyl maltoside in assay buffer without EDTA as described by Duzgunes *et al *[35]. Fluorescence measurements were carried out at 25°C using a Molecular Devices SpectroMAX GeminiEM spectrofluorometer. The extent of vesicles aqueous content mixing was determinated according to the following equation:

Where F_0 _is the value of initial fluorescence of the vesicles, F_t _is the value of fluorescence after incubation for t minutes with the protein, and F_max _is the value of fuorescence after addition of 0.2% of n-dodecyl maltoside.

### Immunoblot analysis

Polyclonal anti-YqiC primary antibodies were obtained in mice immunized with purified YqiC. Immobilon-NC Transfer Membranes (Millipore) containing transferred proteins were blocked in 5% nonfat milk PBS for 1 h, and incubated with either a 1:200 dilution of polyclonal anti-YqiC or 1:200 anti-MBP mouse polyclonal antibodies. The secondary antibody used was goat anti-mouse IgG (Fc Specific) Peroxidase Conjugate (Sigma) at 1:1000 dilution. Positive signals were detected with Chemiluminiscent ECL Plus Western Blotting Detection System (Amersham Biosciences) on a Storm Image and Detection system (Molecular Dynamics).

### Cell fractionation

Wild-type S. Typhimirium strain was grown in 80 mL LB medium to an OD600 of 1 and harvested by centrifugation at 4000 × g. The pellet was resuspended in 3 ml 20 mM Tris-HCl (pH 8.0) and 150 mM NaCl and mechanically lysed in a FastPrep instrument. Cell debris was removed by centrifugation for 30 min at 8000 × g. Subsequently, membranes were sedimented by ultracentrifugation for 1 h at 100,000 × g (4°C). The pellet was resuspended in a volume equivalent to that of the supernatant. Samples from the supernatant and pellet fraction were analyzed by immunoblotting.

### Construction of yqiC *S. Typhimurium *mutant strain

Elimination of the *yqiC *gene was achieved by using Lambda Red-mediated recombination described previously [36]. A lineal DNA fragment that contains the sequence for a chloramphenicol resistance cassette plus flanking regions of *yqiC *was constructed by PCR using the pair of primers 5'-CGCACTACAATAAGAGCTAACACTTACCAGTTCAGGGAAAGTGTAGGCTGGAGCTGCTTCG-3' and 5'-TGGATCGACTGGCGGAATGGCGGGCGCAGGTTTTACTTCTCATATGAATATCCTCCTTA-3'. 5 μg of this construction were introduced into strain LB5010 by electroporation. Chloramphenicol resistant colonies were then verified by PCR using a set of primers that hybridize within the insertion cassette and with an adjacent chromosomal region. Finally, isogenic strain was constructed by P22-mediated transduction of the mutant DNA into *S*. Typhimurium ATCC 14028. The substitution of the *yqiC *gene in this strain was verified by PCR and by the lack of expression of YqiC protein using western blot assay. The *S*. Typhimurium Δ*yqiC*::*CAT *mutant was named 14028 Δ*yqiC*::CAT.

### Mice infections

To determine the 50% lethal dose (LD50) of the *S*. Typhimurium strains used, groups of seven 6-8 weeks old, female, BALB/c mice were infected intraperitoneally (i.p.) with serial 10-fold dilutions (from 1 × 10^1 ^to 1 × 10^5 ^CFU) of the wild type *S*. Typhimurium ATCC 14028 or 14028 Δ*yqiC*::CAT, and deaths were recorded for 28 days. For oral infections with *S*. Typhimurium ATCC 14028, 14028 Δ*yqiC*::CAT and 14028 Δ*yqiC*::CAT trans-complemented with pBBR-*yqiC*, mice were starved for food and water for 4 h. Following starvation, 10^5 ^CFU of each specific strain in 100 μl of phosphate-buffered saline (pH 7.4) were administered by oral gavage to each mouse. Survival of infected mice was recorded over 30 days. Inoculation doses were verified by serial dilution and plating into LB agar.

### Cell invasion and intracellular replication

J774 murine macrophages and HeLa human epithelial cell lines were seeded at a density of 2 × 10^5 ^cells per well in 24-well culture plates. Stationary phase cultures of *S*. Typhimurium ATCC 14028, 14028 Δ*yqiC*::CAT and complemented strain 14028 Δ*yqiC*::CAT + pBBR-*yqiC *grown at 28°C overnight were added to the cells at a multiplicity of infection (MOI) of 10. Culture plates containing infected cells were centrifuged at 1000 rpm for 10 min and incubated at 37°C for 30 min to allow bacterial uptake and invasion. The extracellular bacteria were removed by washing thrice with PBS and incubating with 100 μg/ml gentamycin for 1 h. Thereafter, the cells were incubated with 25 μg/ml gentamycin for the rest of the experiment. After 1, 6 and 24 h, the cells were lysed with 1 mL of 0.1% Triton-X 100 per well and bacterial counts were determined by plating serial dilutions of the lysates on LB agar plates with appropriate antibiotic followed by incubation at 28°C.

## Authors' contributions

MCC, CPO, GAV and AA carried out the molecular biology, protein studies, mice experiments and participated in the draft of the manuscript. GFA, GVE and CSL conceived the study and participated in its design and coordination and drafted the manuscript. All authors read and approved the final manuscript.
